# Assessment of two DNA extraction kits for profiling poultry respiratory microbiota from multiple sample types

**DOI:** 10.1371/journal.pone.0241732

**Published:** 2021-01-06

**Authors:** Michael E. C. Abundo, John M. Ngunjiri, Kara J. M. Taylor, Hana Ji, Amir Ghorbani, Mahesh K. C., Bonnie P. Weber, Timothy J. Johnson, Chang-Won Lee

**Affiliations:** 1 Food Animal Health Research Program, Ohio Agricultural Research and Development Center, The Ohio State University, Wooster, Ohio, United States of America; 2 Department of Veterinary Preventive Medicine, College of Veterinary Medicine, The Ohio State University, Columbus, Ohio, United States of America; 3 Department of Veterinary and Biomedical Sciences, University of Minnesota, Saint Paul, Minnesota, United States of America; 4 Mid-Central Research and Outreach Center, University of Minnesota, Willmar, Minnesota, United States of America; USDA-Agricultural Research Service, UNITED STATES

## Abstract

Characterization of poultry microbiota is becoming increasingly important due to the growing need for microbiome-based interventions to improve poultry health and production performance. However, the lack of standardized protocols for sampling, sample processing, DNA extraction, sequencing, and bioinformatic analysis can hinder data comparison between studies. Here, we investigated how the DNA extraction process affects microbial community compositions and diversity metrics in different chicken respiratory sample types including choanal and tracheal swabs, nasal cavity and tracheal washes, and lower respiratory lavage. We did a side-by-side comparison of the performances of Qiagen DNeasy blood and tissue (BT) and ZymoBIOMICS DNA Miniprep (ZB) kits. In general, samples extracted with the BT kit yielded higher concentrations of total DNA while those extracted with the ZB kit contained higher numbers of bacterial 16S rRNA gene copies per unit volume. Therefore, the samples were normalized to equal amounts of 16S rRNA gene copies prior to sequencing. For each sample type, all predominant bacterial taxa detected in samples extracted with one kit were present in replicate samples extracted with the other kit and did not show significant differences at the class level. However, a few differentially abundant shared taxa were observed at family and genus levels. Furthermore, between-kit differences in alpha and beta diversity metrics at the amplicon sequence variant level were statistically indistinguishable. Therefore, both kits perform similarly in terms of 16S rRNA gene-based poultry microbiome analysis for the sample types analyzed in this study.

## Introduction

The importance of gut and respiratory microbiota in poultry health and production performance has resulted in an increase of studies aimed towards developing microbiome-based intervention strategies [1–5]. Consequently, different protocols for sampling, sample processing, DNA extraction, sequencing, and bioinformatic analysis have emerged. The varying protocols utilized by different research groups can introduce confounding factors and complicate data comparison between studies. For example, the variation attributed to different protocols and its effect on biodiversity profiles in human gut and environmental microbiota has been widely reported [6–14]. We and others have evaluated the sample collection methods for poultry respiratory and gut microbiota [2, 15–17]. However, confounding factors associated with DNA extraction, sequencing strategies, and bioinformatic analysis in poultry microbiota remain to be studied.

An investigation carried out by the Microbiome Quality Control Project, where identical sets of samples were processed in 15 laboratories, identified the DNA extraction process as a significant cause of variation between laboratories, only second to sample type and origin [7]. Efficient and bias-free bacterial DNA extraction for microbiota analysis requires effective disruption of the diverse cell wall structures and compositions present in different types of bacteria [18]. Kits for bacterial DNA extraction are designed to disrupt cell walls mostly through mechanical, chemical, and enzymatic methods [19]. Each of these lysis methods has its own advantages and disadvantages. While mechanical lysis can disrupt all types of bacteria and increase DNA yield, it can shear or fragment genomic DNA and compromise the outcomes of downstream applications, including sequencing [20, 21]. Enzymatic and chemical lysis methods are less likely to damage DNA, but their inefficiency in disrupting certain types of bacteria may introduce bias in the biodiversity profiles [22]. Another important consideration for choosing extraction kits are the contaminating DNAs that are ubiquitously present in kits and reagents. Kit contaminants, especially nucleotide contaminants, critically impact the biodiversity results obtained from low biomass samples [23, 24].

Five different DNA extraction kits were previously used in poultry respiratory microbiota studies, including BiOstic FFPE Tissue DNA Isolation Kit, PowerSoil DNA Isolation Kit, QIAmp DNA Mini Kit, Maxwell RSC PureFood Pathogen Kit, and Qiagen DNeasy Blood and Tissue Kit [2, 15–17, 25–28]. These kits typically employ a combination of mechanical, chemical, and enzymatic disruption methods to increase DNA yields. In our previous poultry respiratory microbiota studies [2, 15–17], the Qiagen DNeasy Blood and Tissue kit (BT kit), which utilizes chemical and enzymatic lysis, was used. The BT kit uses proteinase K, a broad-spectrum serine protease, that can potentially lyse all types of bacteria, but an optional use of lysozyme can ensure complete lysis of gram-positive bacteria. However, the BT kit is not certified for the extraction of low biomass samples, such as respiratory tract swabs, without the use of carrier nucleic acids, which can complicate downstream applications [29].

In this study, we compared the biodiversity profiles of chicken respiratory microbiota generated from DNA samples extracted using the BT kit and ZymoBIOMICS DNA Miniprep kit (ZB kit). The ZB kit uses a combination of mechanical and chemical disruption methods and is a certified low bioburden kit (guaranteed to contain < 3 contaminating bacterial background genomic copy per μL of eluate) [30]. Pre-sequencing results showed great differences between the BT and ZB kits in DNA yield and 16S rRNA gene copies per unit volume. However, after sequencing of normalized samples, both kits produced similar microbiome data in terms of bacterial diversity at the amplicon sequence variant (ASV) level and taxonomic profiles at the class and genus levels.

## Materials and methods

### Animals and ethics statement

Animal management and euthanasia were in correspondence with protocol #2015A00000056 approved by The Ohio State University Institutional Animal Care and Use Committee. This protocol conforms with the U.S Animal Welfare Act, Guide for Care and Use of Laboratory Animals and Public Health Service Policy on Humane Care and Use of Laboratory Animals. The Ohio State University is accredited by the Association for the Assessment and Accreditation of Laboratory Animal Care International. White leghorn chickens were obtained from our institution’s (Food Animal Health Research Program, Wooster, OH) specific-pathogenfree poultry flock [17]. The chickens were provided with *ad libitum* access to feed and water and their welfare was monitored twice daily. All birds were clinically healthy and were all included in the study. Prior to the collection of invasive samples, the birds were humanely euthanized through carbon dioxide exposure as previously described [17].

### Experimental design and sample collection

**Fig 1:**
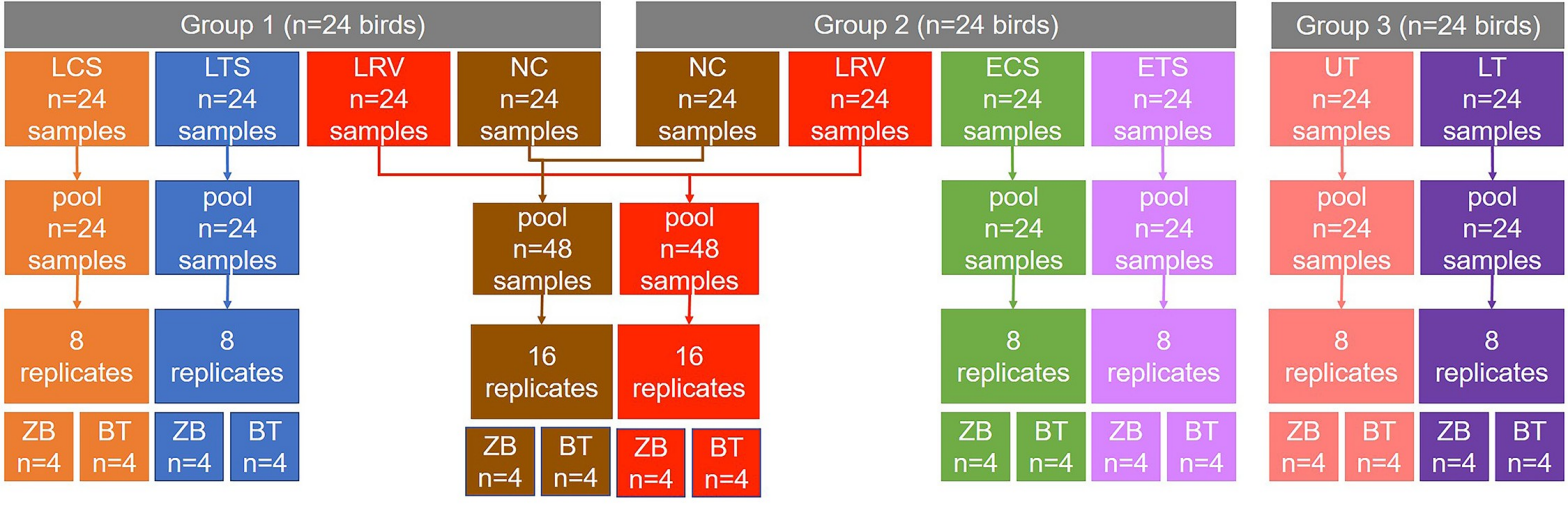
Experimental design. A total of 72 four-week-old SPF chickens were divided into three groups on the day of sampling. Samples were collected from individual birds and pooled as shown above. Each sample pool was aliquoted into several replicates that were evenly divided between the BT and ZB kits. LCS, live choanal swab; ECS, euthanized choanal swab; LTS, live tracheal swab; ETS, euthanized tracheal swab; NC, nasal cavity wash; UT, upper trachea wash; LT, lower trachea wash; LRV, lower respiratory lavage.

A total of 72 four-week-old specific-pathogen-free chickens were hatched in the same incubator, then co-housed together for four weeks prior to sampling. At the day of sampling, the birds were randomly assigned to three groups for sample collection based on the target sample types and sampling techniques (Fig 1). The sample size was decided based on our recent reports showing that four or more respiratory samples were sufficient for 16S rRNA gene-based taxonomic and diversity analysis [17]. An assortment of invasive and non-invasive respiratory samples including, nasal cavity wash, upper and lower tracheal wash, lower respiratory lavage, as well as choanal and tracheal swabs, were obtained from either live or euthanized birds. For the collection of tracheal and choanal swab samples, the swabs were gently inserted into the trachea or choanal cleft, moved 5 times along the cleft or the trachea, and then subsequently immersed in 1 mL of 1X PBS. To collect tracheal washes, the upper and lower tracheal tissues were aseptically excised, stored in a sterile tube, then immediately snap-frozen in liquid nitrogen. Each frozen trachea was thawed, and the luminal mucosa was washed using 1X PBS in a biosafety cabinet. For the collection of nasal cavity wash, the outer nares were thoroughly wiped with a cotton ball containing 100% ethanol to remove any visible organic material. The nasal cavity was then flushed through the nostrils with 1X PBS, until a total of approximately 1 mL of nasal wash was collected from each bird. For the collection of lower respiratory lavage, a 25 mL syringe containing 1X PBS with a fitted pipet tip was used to wash the lower respiratory tract until a total of 40–45 mL of lavage was collected. The samples were pooled as follows: 24 samples per pool for tracheal swabs from live birds, choanal swabs from live birds, tracheal swabs from euthanized birds, choanal swabs from euthanized birds, upper tracheal wash pellets, lower tracheal wash pellets; and 48 samples per pool for nasal cavity wash pellets and lower respiratory lavage pellets. Pooling of the lower respiratory lavage and nasal cavity wash samples depended on whether the trachea was to be excised, or not. Birds in group 3 could not be used for lower respiratory lavage for practical reasons, since the entire trachea was excised leaving no place to attach the lavage collection apparatus. On the other hand, in groups 1 and 2, the trachea was cut at about 5 mm above the syrinx to support the attachment of the apparatus. Likewise, the nasal cavity wash was collected only from swabbed birds after swabbing, to prevent cross contamination between the nasal cavity and the trachea, since the procedure of washing the nasal cavity can have some leakage to the trachea. The pooled samples were homogenized and divided into 8 replicates for tracheal and choanal swabs and tracheal wash pellets, and into 16 replicates for the nasal cavity wash pellets and lower respiratory lavage pellets. The sample pools were evenly divided to be extracted with either the BT or ZB kit. This study did not involve treatment groups and, therefore, no control animals were required.

### Sample processing and DNA extraction

All the respiratory samples were pelleted by centrifugation at 10,000 × g at 4°C for 10 mins. After the supernatant was removed, the pellet was resuspended and divided accordingly into replicates (Fig 1).

The general steps for BT kit extraction were as follows: all the respiratory samples were pretreated for gram-positive bacteria using an enzymatic lysis buffer containing 20 mM Tris·Cl, pH 8.0, 2 mM sodium EDTA, 1.2% Triton X-100, and 20 mg/mL of lysozyme. After incubation time (3 hours for nasal cavity wash and swab samples, 45 min for the trachea wash and lower respiratory lavage samples) at 37°C, 100% ethanol was added. The mixture was then added to a spin column for DNA isolation, and each column was washed with buffers AW1 and AW2, respectively. Finally, 50 μL of elution buffer AE was used to elute DNA from the spin column membrane.

Since our samples were collected from multiple respiratory sites, subtle modifications to the BT kit extraction protocol were implemented. For extraction of nasal cavity wash, tracheal swab, and choanal swab samples, the manufacturer’s protocol was modified to include the lysozyme-based enzymatic lysis buffer option for disruption of gram-positive bacteria and increasing the lysis incubation time from 30 min to 3 hours (MBT-A protocol) [29]. The longer incubation time was implemented due to the high levels of organic material in these samples [17]. Upper tracheal wash, lower tracheal wash, and lower respiratory lavage samples were extracted using another modified protocol where the enzymatic lysis incubation time was increased from 30 min to 45 min (MBT-B protocol) as previously described [17]. In the MBT-A protocol, proteinase K, Buffer AL, and 100% ethanol volumes were as recommended by the manufacturer’s protocol, while AW1 and AW2 Wash Buffer volumes were increased from 500 μL to 730 μL. The volumes of these reagents were increased 5 times in the MBT-B protocol to improve DNA quantities and purity as previously described [17]. In both protocols, DNA was eluted using 50 μL of elution Buffer AE, reapplied in the column, incubated for 5 min, and eluted once more.

Extraction of DNA from respiratory samples using the ZB kit was performed as per the manufacturer’s direction [30]. Identical ZB kit extraction protocol was implemented regardless of the sample type. The steps for the ZB kit extraction were as follows: initially, 730 μL of lysis solution and beads (size = 0.1 & 0.5 mm) were added to the respiratory pellets. The tubes were fastened to a Disruptor Genie vortex (Scientific Industries, Bohemia, NY) using a vortex adapter. The samples were vortexed at maximum speed for 20 min. The samples were then centrifuged at 8,000 × g for 1 min, and 400 μL of supernatant was added to III-F filter and centrifuged once more at 8,000 × g for 1 min. Afterwards 1,200 μL of DNA binding buffer was added to the filtrate and the mixture was transferred to IICR Column and centrifuged at 10,000 × g for 1 min. Once the flow-through was discarded, the column was washed with 400 μL of Wash Buffer 1 and 700 μL of Wash Buffer 2, centrifuged at 10,000 × g, and the flow-through was discarded. Next, an additional 200 μL of Wash Buffer 2 was added to the column and centrifuged for 10,000 × g for 1 min. For the elution process, 50 μL of DNase/RNase free water was directly added to the column, incubated for 1 min, and centrifuged at 16,000 × g for 3 min. As a final step, the DNA eluate was passed through the III-HRC prepped filter and centrifuged at 16,000 × g for 3 min.

Both a mock community control (ZymoBIOMICS Microbial community standard) and negative extraction controls (1X PBS) were extracted alongside the samples in both kits. At least two negative extraction controls were collected with each sample type for each kit. The negative extraction controls were subjected to each step, all the way from sample collection to 16S rRNA gene sequencing.

### 16S rRNA gene sequencing

Our optimized methods for pre-sequencing and sequence processing are detailed in our recent reports [15–17]. Briefly, DNA concentration was quantified using NanoDrop 2000c spectrophotometer (Thermo Fisher Scientific, Waltham, MA) and adjusted to a concentration of 100 ng/μL. Next, 16S rRNA genes in the DNA samples were PCR-amplified and sequenced at the University of Minnesota Genomics Center. The 16S rRNA gene copies in the samples were determined by using quantitative PCR (qPCR) of the V4 hypervariable region using primer sets 515F (5′-TCGTCGGCAGCGTCAGATGTGTATAAGAGACAGGTGCCAGCMGCCGCGGTAA-3′) and 806R (5′-GTCTCGTGGGCTCGGAGATGTGTATAAGAGACAGGGACTACHVGGGTWTCTAAT-3′) Nextera primers [31]. The conditions for qPCR were as follows: one cycle of 95°C for 5 min; followed by 35 cycles of 98°C for 20 s, 55°C for 15 s, and 72°C for 1 min; and one cycle of 72°C for 5 min using QuantStudio Real-Time PCR System (Thermo Fisher Scientific, Waltham, MA). The Cycle Threshold (CT) values were then converted to 16S rRNA gene copy numbers using a standard curve. The samples were then normalized to 1.67 × 10^5^ 16S rRNA gene copies/μL then subjected to two rounds of PCR. In the first round, 515F and 806R Nextera primers were used for the amplification of V4 region from 3 μL of normalized DNA using the following cycling parameters: one cycle of 95°C for 5 min, followed by 25 cycles of 98°C for 20 s, 55°C for 15 s, and 72°C for 1 min. The PCR product was then diluted to 1:100, and 5 μL was used in the second round of PCR using forward (5′-AATGATACGGCGACCACCGAGATCTACAC(i5)TCGTCGGCAGCGTC-3′) and reverse (5′-CAAGCAGAAGACGGCATACGAGAT(i7)GTCTCGTGGGCTCGG-3′) indexing primers (Integrated DNA Technologies, Coralville, IA). In the second PCR, the following cycling parameters were used: 1 cycle at 95°C for 5 min, followed by 10 cycles of 98°C for 20 s, 55°C for 15 s, and 72°C for 1 min. Pooled size-selected samples were denatured using NaOH, diluted to 8 pM in Illumina’s HT1 buffer, spiked with 20% PhiX, and heat denatured at 96°C for 2 min immediately before loading. MiSeq 600 (2 × 300 bp) cycle v3 kit (Illumina, San Diego, CA) was used to sequence the samples. This protocol is an optimization of the Earth Microbiome Project protocol [32, 33].

### Data processing and statistical analysis

Fastq files for each sample were generated after sequence demultiplexing. BBTools (v35), a suite of DNA/RNA analysis software provided by the Joint Genome Institute, was used to trim both proximal and distal primers and adapters from the sequences using the BBDuk module, and paired-end reads were merged using the BBMerge module [34]. Cutadapt (v1.4.2) was used to remove 16S rRNA gene amplicon primers [35]. The BBMap module of BBTools were used to filter sequence lengths of less than 245 and greater than 260 base pairs [34].

QIIME 2 (qiime2-2019.1) and software implemented therein, were used for additional data processing and statistical analysis [36]. Using a Casava 1.8 single-end demultiplexed format, sequences were imported into QIIME 2. DADA2, pooled chimera filtering method, was used to denoise the sequences [37]. VSEARCH [38] was used for chimeric sequence detection, non-16S rRNA gene identification, and open reference clustering of amplicon sequence variants (ASVs) with a similarity threshold of 100% using the SILVA (release 132) reference database [39]. Furthermore, ASVs with a total frequency of < 10 in the entire table and present in only 1 sample were removed. In addition, to generate a feature table using MAFFT, a de novo alignment of the representative set of sequences was used, which eliminated non-conserved and highly gapped alignment columns by applying the 'mast' option of the alignment plugin. The aligned filtered sequences were used to reconstruct a phylogenetic tree using Fast Tree, that was then rooted at the mid-point prior to beta diversity analysis.

A Naïve Bayes classifier was trained using the 16S rRNA gene sequences covered in the 515F (GTGYCAGCMGCCGCGGTAA) and 806R (GGACTACNVGGGTWTCTAAT) primer pair region for taxonomic classification [40]. For the respiratory samples, the classifier was trained on the reference sequences derived from the SILVA 16S rRNA gene database (release 132) based on matches with this primer pair and a taxonomic identification table (majority classification) based on 99% identity clustered reference sequences. For the mock community samples, the classifier was trained using the 16S rRNA sequences provided by ZymoBIOMICS with identical parameters as described above. Finally, the classifier was used in our experimental data to assign taxonomy to the observed ASVs. 5000 reads per sample was the rarefaction cut-off in the final feature table [15–17].

Weighted and unweighted UniFrac distance metrics were used to assess beta diversity variations in the bacterial community structure between extraction kits [41]. Statistical differences in the beta diversity for each sample type between the kits were quantified by pairwise permutational multivariate analysis of variance (PERMANOVA) with a Benjamini-Hochberg correction for false discovery [42]. The number of observed ASVs [43] was used for alpha diversity analysis to evaluate the richness of bacterial species for each sample, and Pielou’s evenness [44] was used to evaluate the relative evenness of species richness. Statistical differences between DNA quality and quantity, 16S rRNA gene copies in total DNA per sample, number of 16S rRNA gene sequences per sample, avian mitochondria sequences per sample, and alpha diversity were examined using a nonparametric Kruskal-Wallis test with Benjamini-Hochberg correction for false discovery [45]. Differences were considered significant at p < 0.05.

Additionally, differentially abundant analysis (DESeq2) and contamination removal (decontam) were conducted in R [46]. DESeq2 was used to detect statistically significant differentially abundant taxa between kits for each respiratory sample type [47]. Adjusted p values < 0.05 (Wald statistic, with a Benjamini-Hochberg correction for false discovery) were considered to be significant [47]. Decontam frequency method (version 1.2.1) was used to identify taxa with significant inverse correlations with 16S rRNA gene concentration as measured via qPCR [48]. Moreover, respiratory samples were filtered based on the number of 16S rRNA gene copies found in the negative extraction control. Samples that contained lower 16S rRNA gene copies compared to the respective negative extraction controls were removed from analysis.

## Results

### BT and ZB kits differ greatly in DNA yield and 16S rRNA gene copy numbers

**Fig 2:**
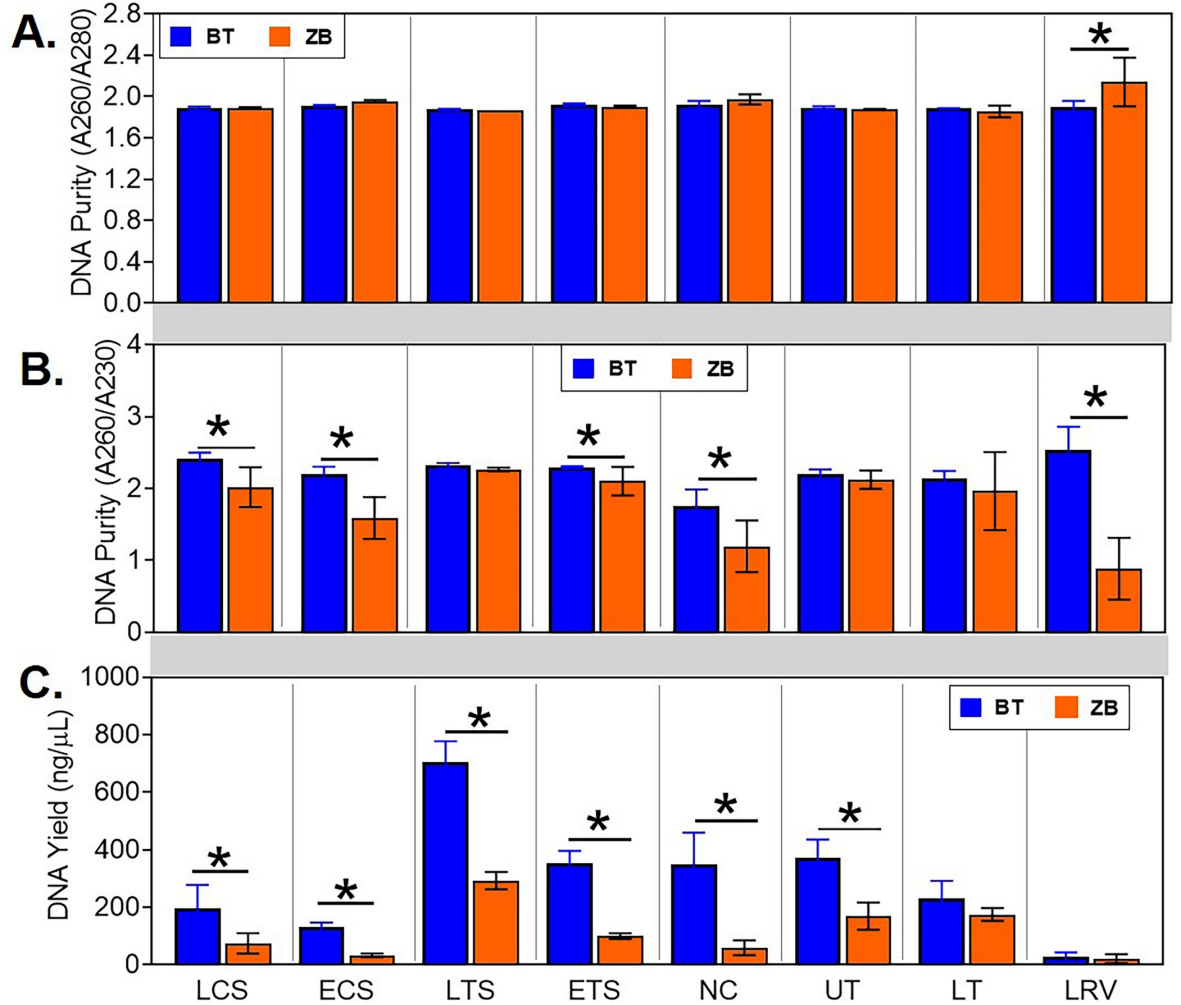
Comparison of quantity and quality of DNA from samples extracted using the ZB and BT kits. The elution volume was standardized to 50 μL for both kits. DNA quality was expressed as ratios of absorbances at (A) 260nm and 280nm wavelengths (A260/A280) and (B) 260nm and 230nm wavelengths (A260/A230). (C) DNA yield was expressed as mass per unit volume (ng/μL). Statistical differences, * p < 0.05 (pairwise Kruskal-Wallis tests, with a Benjamini-Hochberg correction for false discovery). LCS, live choanal swab; ECS, euthanized choanal swab; LTS, live tracheal swab; ETS, euthanized tracheal swab; NC, nasal cavity wash; UT, upper trachea wash; LT, lower trachea wash; LRV, lower respiratory lavage.

For both kits, DNA was eluted in a volume of 50 μL to allow between-kits comparison of DNA concentrations and purity. DNA quality was spectrophotometrically measured and expressed as ratios of absorbances at 260 nm and 280 nm wavelengths (A260/A280) and 260 nm and 230 nm wavelengths (A260/A230). The mean A260/A280 values for most of the samples were around 1.8 and were not statistically different between the kits. However, the mean value for the lower respiratory lavage samples extracted with the ZB kit was higher than 2.0 which was significantly higher compared to the BT kit (Fig 2A). On the other hand, higher mean A260/A230 ratios were observed for most of the samples extracted using the BT kit, with significantly higher ratios in the live choanal swab, euthanized choanal swab, euthanized tracheal swab, nasal cavity wash, and lower respiratory lavage (Fig 2B). DNA yield was spectrophotometrically measured and expressed as mass per unit volume (ng/μL). In general, the BT kit tended to have more DNA yield than the ZB kit, with DNA quantities being significantly higher for most of the sample types except for the lower trachea wash and lower respiratory lavage (Fig 2C).

The DNA was subsequently subjected to qPCR to determine the number of 16S rRNA gene copies. Negative extraction controls were included to facilitate filtering out of samples with low amounts of 16S rRNA gene copies. The highest amounts of 16S rRNA gene copies detected in the negative controls were set as elimination thresholds for the corresponding sample types. The elimination cut-offs for the BT kit were 1.6 × 10^3^, 1.4 × 10^4^, 2.0 × 10^3^, 3.1 × 10^3,^ and 1.1 × 10^4^ 16S rRNA gene copies per nanogram of total DNA for choanal swab, lower respiratory lavage, nasal cavity wash, tracheal swab, and lower and upper tracheal washes, respectively. The elimination cut-offs for the ZB kit were 20, 50, 20, 60, and 15 16S rRNA gene copies per nanogram of DNA for choanal swab, lower respiratory lavage, nasal cavity wash, tracheal swab, and tracheal wash, respectively. Using these criteria, 3 out of the 40 samples extracted using the BT kit were dropped from further analysis (1 live tracheal swab and 2 lower respiratory lavage samples). In contrast, all 40 samples extracted using the ZB kit were retained.

**Fig 3:**
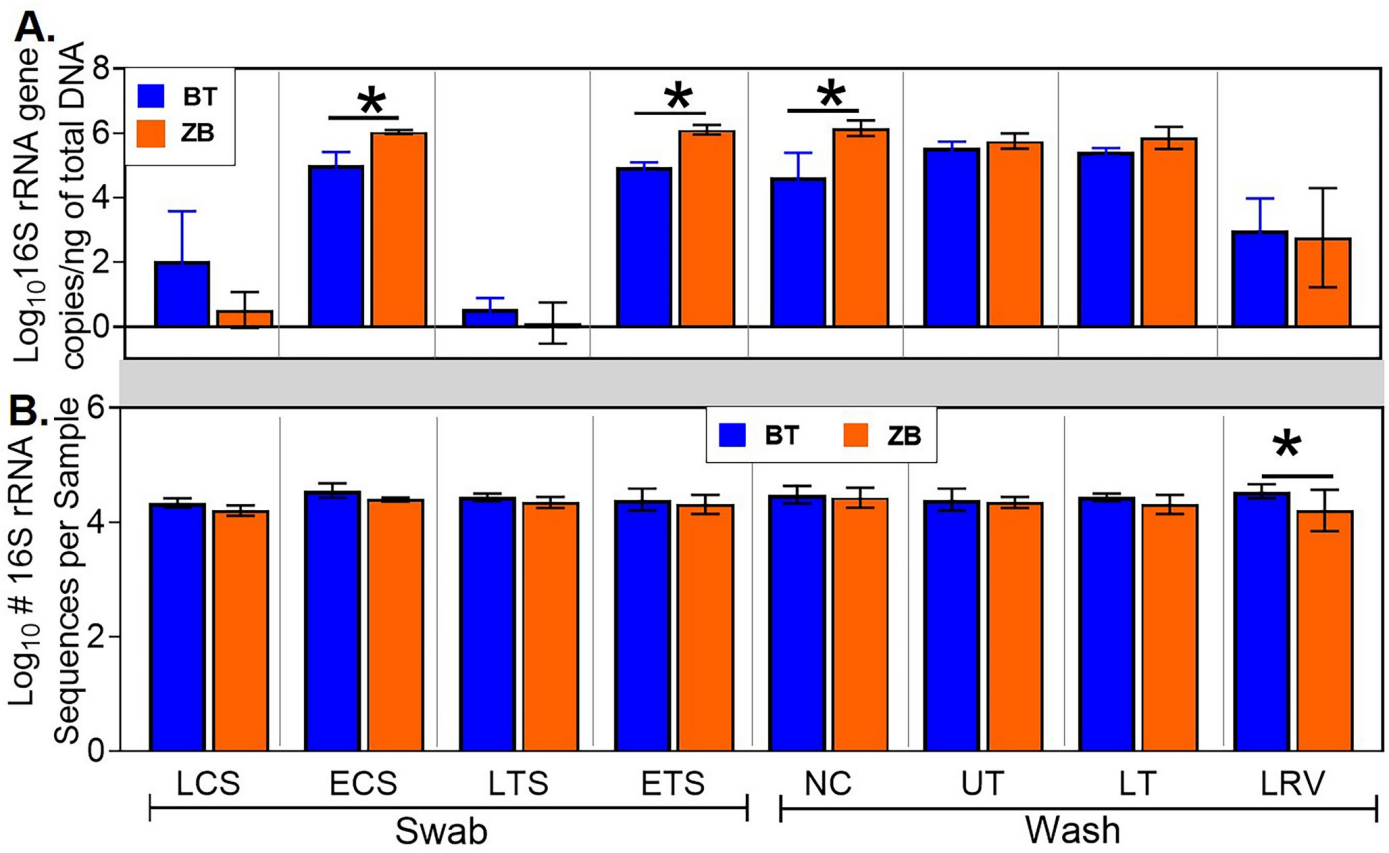
Between-kit comparison of pre- and post-sequencing 16S rRNA gene copies for each sample type. (A) Pre-sequencing 16S rRNA gene copies per nanogram of total DNA. Quantitative PCR was used to determine the number of 16S rRNA gene copies per μL of sample. The DNA samples were then normalized to 1.67 × 10^5^ 16S rRNA gene copies/μL for subsequent PCR prior to sequencing. (B) Post-sequencing 16S rRNA gene sequence counts. The sequences were quality-filtered as described in Materials and methods. Statistical differences, * p < 0.05 (pairwise Kruskal-Wallis tests, with a Benjamini-Hochberg correction for false discovery). LCS, live choanal swab; ECS, euthanized choanal swab; LTS, live tracheal swab; ETS, euthanized tracheal swab; NC, nasal cavity wash; UT, upper trachea wash; LT, lower trachea wash; LRV, lower respiratory lavage.

**Fig 4:**
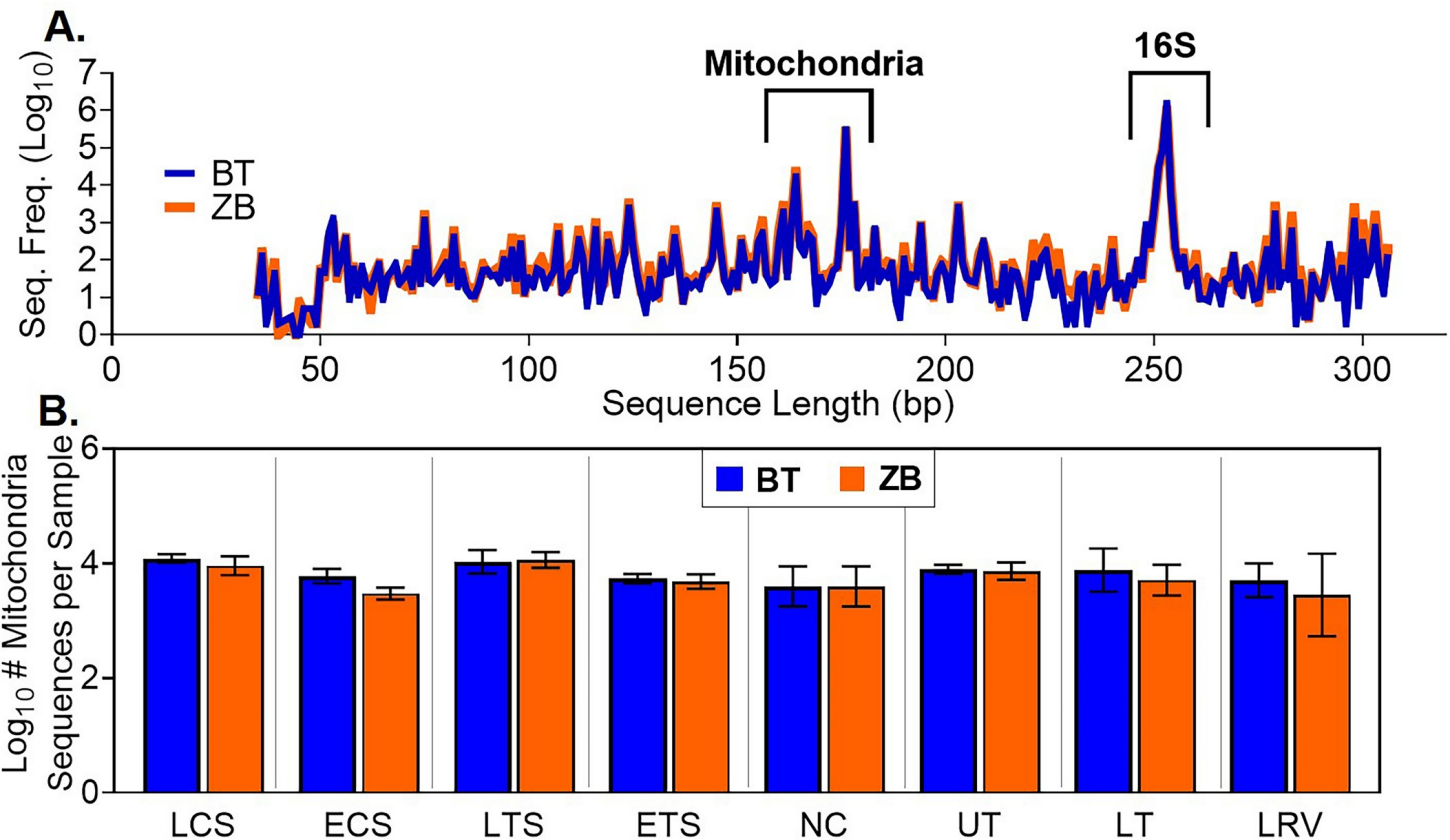
Host sequences detected in Illumina sequencing data. (A) Sequence length distribution showing the size ranges for avian mitochondria (175, 176, 178, and 194 bp) and 16S rRNA gene sequences (250–264 bp). (B) Statistical comparisons of mitochondria gene sequences in different tissue types. Statistical differences, * p < 0.05 (pairwise Kruskal-Wallis tests, with a Benjamini-Hochberg correction for false discovery).

Among the retained samples, the nasal cavity wash, euthanized tracheal swab, and euthanized choanal swab samples extracted with the ZB kit had significantly higher numbers of 16S rRNA gene copies than those extracted using the BT kit (Fig 3A). Subsequently, all samples were normalized to 1.67 × 10^5^ 16S rRNA gene copies/μL. This was done to achieve 5 × 10^5^ 16S rRNA gene copies in 3μL of normalized sample used for pre-sequencing PCR, which approximately translates to a target sequencing coverage of at least 10X. The PCR products were subsequently used for sequencing library preparation. No significant differences in the 16S rRNA gene sequence counts from the normalized samples were observed between the kits, except for the lower respiratory lavage samples (Fig 3B). Additionally, a substantial number of non-16S rRNA gene sequences, including host avian mitochondria gene sequences, were detected in samples extracted with either kit (Fig 4). Avian mitochondria gene sequences were 175, 176, 178, and 194 bp in length, while the 16S RNA gene sequence lengths were between 250–264 bp (Fig 4A). For all sample types, no significant differences were detected in the number of avian mitochondria gene sequences between the kits (Fig 4B).

### Minimal contaminants were identified, and the kits displayed subtle differences in extraction efficiencies

Several bacterial taxa were found in the negative extraction controls, indicating the presence of contaminants originating from either the reagents, sample processing steps, or sequence library preparation. These taxa were analyzed using decontam R package to identify contaminants in the respiratory samples [48]. Of the 1487 ASVs detected among the samples, 14 were identified to be contaminants (Table 1). The contaminants were detected in low abundances (< 0.15% relative abundance) independently of the kit used for extraction.

**Table 1:** Contaminants identified in negative and mock community controls.

	Negative		Mock Community	
	BT	ZB	BT	ZB
*Acetobacteroides*	0.004*	0.000		
*Acinetobacter*	0.004	0.000		
*Bacillus*	0.000	0.034		
*Bacteroides*			0.011	0.000
*Candidatus Woesebacteria*	0.004	0.000		
Clostridiales	0.004	0.006		
*Clostridium sensu stricto* 1	0.004	0.000		
*Corynebacterium* 1	0.020	0.138		
*Faecalibacterium*			0.003	0.007
*Flavobacterium*			0.003	0.000
*Halomonas*			0.033	0.026
*Lactobacillus*			0.090	0.057
*Lactobacillus vaginalis*			0.000	0.026
*Phascolarctobacterium*	0.000	0.052		
*Phyllobacterium*	0.004	0.006		
*Polymorphobacter*	0.004	0.000		
*Pseudoalteromonas*			0.003	0.000
*Pseudochrobactrum*			0.000	0.009
*Ruminiclostridium*	0.004	0.000		
Ruminococcaceae	0.004	0.006		
*Sphingomonas*			0.029	0.037
*Stenotrophomonas acidaminiphila*	0.004	0.000		
*Streptococcus*			0.004	0.000
*Streptococcus varani*			0.000	0.004
uncultured bacterium	0.004	0.000		
*Vibrio*			0.141	0.143
**Total abundance**	**0.064**	**0.242**	**0.318**	**0.31**

*Values represent relative abundance as a percentage of pooled sequences from all sample types extracted with the indicated kit.

A mock community control was processed alongside the respiratory samples to assess the efficiency of extraction between the kits and the extent of contamination during the extraction process. Contaminants in the mock community samples were identified based on their absence in the compositional shotgun sequence data provided by ZymoBIOMICS in the certificate of analysis [49]. Consistent with our previous findings [17], the mock community had < 1% contaminant bacteria (Table 1). While all bacteria in the mock community were detected in samples extracted with either kit, their relative abundances based on 16S rRNA gene sequencing varied compared to the theoretical 16S rRNA gene abundances (Table 2) [50]. Even though disparities in the relative abundance of 16S rRNA gene sequences were detected between the kits, only *Listeria monocytogenes* and *Staphylococcus aureus*, both gram-positive bacteria, were found to be significantly higher in the ZB kit as detected using DESeq 2 [47]. However, this difference may be due to the low number of mock community replicates available for this study (Table 2).

**Table 2:** Efficiency of mock community extraction.

Mock Community Taxonomic Identity	Gram stain	Relative Abundance of 16S rRNA gene sequences (%)		
		BT (n = 3)	ZB (n = 2)	Theoretical^†^
*Bacillus subtilis*	+	15.6 ± 1.4	16.3 ± 0.8	17.4
Listeria monocytogenes*	+	4.3 ± 1.2	9.9 ± 0.9	14.1
Staphylococcus aureus*	+	6.5 ± 0.7	15.4 ± 4.5	15.5
*Enterococcus faecalis*	+	8.6 ± 1.1	7.5 ± 0.3	9.9
*Lactobacillus fermentum*	+	25.6 ± 0.4	21.8 ± 1.2	18.4
*Escherichia coli*	-	13.3 ± 0.2	10.3 ± 2.4	10.1
*Salmonella enterica*	-	13.3 ± 0.6	9.6 ± 3.6	10.4
*Pseudomonas aeruginosa*	-	12.7 ± 0.4	9.1 ± 1.7	4.2

^†^Theoretical 16S values were calculated based on theoretical genomic DNA composition as provided by ZymoBIOMICS for Catalog No: D6300, using the following formula: 16S/18S copy number = total genomic DNA (g) × unit conversion constant (bp/g) / genome size (bp) × 16S/18S copy number per genome. https://files.zymoresearch.com/protocols/_d6300_zymobiomics_microbial_community_standard.pdf.

Statistical differences

* p < 0.05 adjusted p-value (Wald statistic, with a Benjamini-Hochberg correction for false discovery).

### Method of extraction had minimal effects on taxonomic composition and diversity

**Fig 5:**
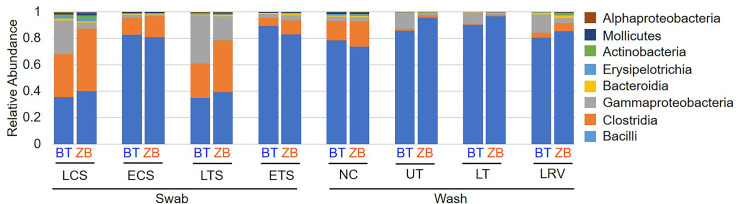
Comparison of relative abundances of different bacteria classes in samples extracted using the BT and ZB kits. LCS, live choanal swab; ECS, euthanized choanal swab; LTS, live tracheal swab; ETS, euthanized tracheal swab; NC, nasal cavity wash; UT, upper trachea wash; LT, lower trachea wash; LRV, lower respiratory lavage.

After filtering the sequence data based on the 16S rRNA gene copy numbers and contaminants in negative extraction controls, the impacts of the extraction kits on basic microbiota parameters were assessed. Both of the extraction kits were able to detect the following predominant taxa at the class level: Bacilli, Clostridia, Gammaproteobacteria, Bacteroida, Erysipelotrichia, Actinobacteria, Mollicutes, and Alphaproteobacteria (Fig 5). Furthermore, several ASVs that were shared between the extraction methods within each sample type constituted > 92% of total abundance in the corresponding bacterial community (Table 3). Although several unique ASVs were detected by either kit, their relative abundances were low (S2 File). In addition, differentially abundant shared taxa were assessed (Table 4). While majority of these differentially abundant taxa had very low relative abundance, *Vibrio* prevalence was highly pronounced in the BT kit.

**Table 3:** Comparison of the number of shared and unique ASVs and their relative abundance between the kits.

Sample type	Shared ASVs			Unique ASVs			
	# of ASVs	Relative Abundance (%)		BT		ZB	
		BT	ZB	# of ASVs	Relative Abundance (%)	# of ASVs	Relative Abundance (%)
Euthanized choanal swab	178	99.42	99.7	99	0.58	59	0.3
Live choanal swab	288	95.15	98.59	151	4.85	123	1.41
Euthanized tracheal swab	128	99.54	99.49	88	0.46	80	0.51
Live tracheal swab	141	92.07	97.76	130	7.93	137	2.24
Nasal cavity wash	285	99.58	99.7	104	0.42	100	0.3
Upper tracheal wash	63	97.67	99.51	72	2.33	72	0.49
Lower tracheal wash	53	98.63	99.59	76	1.37	54	0.41
Lower respiratory lavage	196	98.43	96.9	118	1.57	186	3.1

**Table 4:** Differentially abundant shared taxa.

Differentially Abundant Taxon	DCS		LCS		DTS		LTS		NW		UT		LT		LV	
	BT	ZB	BT	ZB	BT	ZB	BT	ZB	BT	ZB	BT	ZB	BT	ZB	BT	ZB
*Lactococcus*	0.03	0.0	**0.4**	**0.3**	0.0	0.0	0.1	0.1	0.2	0.2	0.0	0.0	0.0	0.0	0.0	0.1
*CHKCI001*	0.02	0.1	**0.1**	**0.4**	0.0	0.2	0.1	0.3	**0.1**	**0.4**	0.0	0.0	NS	NS	0.0	0.1
*[Eubacterium] hallii group*	0.05	0.2	**0.1**	**0.5**	0.0	0.2	0.0	0.2	**0.1**	**0.4**	0.0	0.0	NS	NS	0.1	0.1
*Ruminococcaceae UCG-004*	NS	NS	**0.0**	**0.2**	0.0	0.0	NS	NS	0.0	0.1	NS	NS	NS	NS	0.0	0.0
*Subdoligranulum*	0.17	0.4	1.1	3.0	0.2	0.4	0.4	1.3	**0.7**	**1.3**	0.0	0.1	0.0	0.1	**0.1**	**0.6**
*Ochrobactrum*	0.00	0.0	NS	NS	0.0	0.0	NS	NS	NS	NS	0.1	0.0	NS	NS	**0.1**	**0.0**
*Pseudoalteromonas*	NS	NS	**1.4**	**0.0**	NS	NS	NS	NS	**0.0**	**0.0**	NS	NS	NS	NS	**0.6**	**0.0**
*Vibrio*	**0.85**	**0.0**	**14.4**	**0.0**	**0.7**	**0.0**	**17.2**	**0.1**	**0.9**	**0.0**	**7.8**	**0.0**	**5.3**	**0.1**	**8.4**	**0.1**
*Stenotrophomonas*	NS	NS	**1.2**	**0.0**	NS	NS	NS	NS	NS	NS	**0.6**	**0.0**	NS	NS	**0.6**	**0.0**
*Halomonas*	NS	NS	NS	NS	NS	NS	NS	NS	**0.1**	**0.0**	NS	NS	**0.7**	**0.0**	NS	NS
*Romboutsia*	0.51	1.0	3.0	5.2	0.3	0.8	1.5	2.1	**0.2**	**1.1**	0.2	0.3	0.1	0.3	**0.0**	**0.3**
unclassified Peptostreptococcaceae	0.07	0.2	**0.2**	**0.9**	0.1	0.3	0.1	0.4	**0.2**	**0.7**	0.0	0.1	0.0	0.0	**0.0**	**0.1**
unclassified Enterobacteriaceae	0.16	0.1	**1.4**	**0.2**	0.4	0.6	2.7	2.1	0.2	0.2	0.7	0.2	0.4	0.2	0.7	0.4

Differentially abundant shared taxon within a sample type between kits identified by DESeq2. Numbers in bold formatting represent significant differences between BT and ZB, p < 0.05 adjusted p-value (Wald statistic, with a Benjamini-Hochberg correction for false discovery). Numbers with an additional gray highlight represents relative abundance differences between the kits of about 1% or higher.

Note: NS denotes taxa that is not shared between the kits within a sample type. Additionally, any zero value on the table is a non-zero value if significant digits are increased to > 5 decimal places.

**Fig 6:**
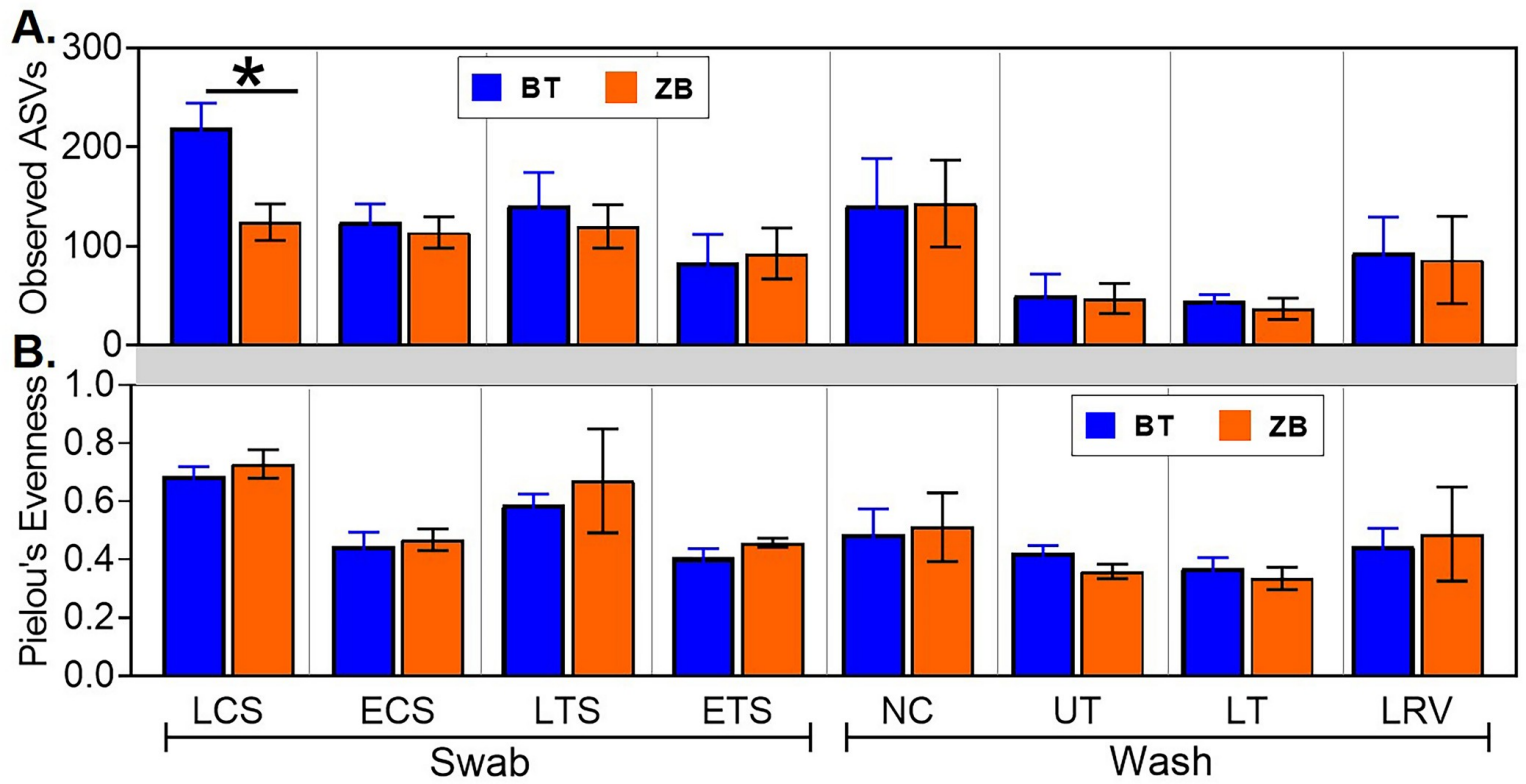
Comparison of alpha diversity metrics from samples extracted using the ZB and BT kits. (A) Species richness was estimated based on the number of observed amplicon sequence variants (ASVs) in each sample. (B) Species evenness in each sample was estimated using Pielou’s evenness index. Statistical differences, * p < 0.05 (pairwise Kruskal-Wallis tests, with a Benjamini-Hochberg correction for false discovery). LCS, live choanal swab; ECS, euthanized choanal swab; LTS, live tracheal swab; ETS, euthanized tracheal swab; NC, nasal cavity wash; UT, upper trachea wash; LT, lower trachea wash; LRV, lower respiratory lavage.

**Fig 7:**
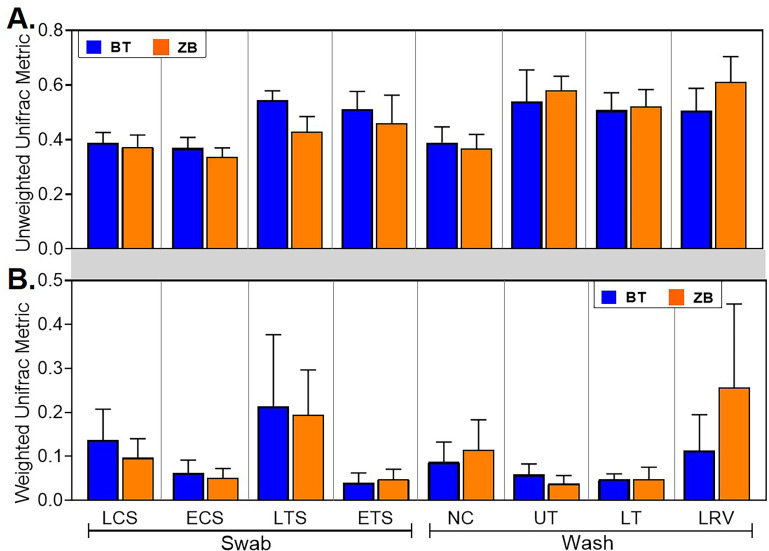
Comparison of beta diversity metrics from samples extracted using the BT and ZB kits. Unweighted (A) and Weighted (B) UniFrac distances were compared between samples from a given sample type. No statistical differences were found between the kits (pairwise permutational multivariate analysis of variance, PERMANOVA, with a Benjamini-Hochberg correction for false discovery). LCS, live choanal swab; ECS, euthanized choanal swab; LTS, live tracheal swab; ETS, euthanized tracheal swab; NC, nasal cavity wash; UT, upper trachea wash; LT, lower trachea wash; LRV, lower respiratory lavage.

The characteristics of the microbial communities detected by each kit were evaluated further through alpha and beta diversity metrics. In majority of the samples, the number of observed ASVs in a given sample type were statistically indistinguishable between the kits, except in the live choanal swab where, on average, samples extracted using the BT kit had a higher number of ASVs (Fig 6A). Similarly, the extraction kit did not affect the species evenness in any of the sample types (Fig 6B). Beta diversity was assessed by comparing within sample type UniFrac distances between kits. No statistical differences were observed between the kits (Fig 7).

## Discussion

The DNA extraction process can be a major source of variation between microbiome studies [7]. This poses a potential problem with inter-study comparison among the growing number of poultry respiratory microbiome studies. Using the BT kit in our previous studies, we were able to characterize the microbial communities present in sinus cavities, tracheas and lower respiratory tracts of turkeys, chicken broilers, and chicken layers raised in commercial settings, and specific-pathogen free chickens raised for research [2, 15–17]. Although we optimized the BT kit for increased DNA yields and removal of PCR inhibitors from respiratory samples [2, 15, 17], the kit is not specifically certified for microbiome sample extraction [29]. Here, we compared the performance of the BT kit with that of the ZB kit, which is validated for extraction of low-bioburden microbiome samples [30]. Overall, while statistical differences were observed between kits in the pre-sequencing results, post-sequencing microbiota data obtained with normalized samples showed only minimal differences between the kits.

Both kits were very efficient in eliminating protein contaminants absorbing at 280 nm as indicated by A260/A280 ratios within the expected range of 1.8–2.0 [29, 51, 52]. However, the ZB kit seemed inefficient at removing non-protein contaminants absorbing at 230 nm from some sample types, especially the nasal cavity wash and lower respiratory lavage samples, as indicated by A260/A230 ratios that were below the expected range of 2.2–2.5 (Fig 2B) [53]. While the exact nature of these impurities remain undetermined, their impact on PCR amplification was negligible as samples extracted with the ZB kit tended to have similar or higher 16S rRNA gene copy numbers compared to those extracted with the BT kit (Fig 3A). Nevertheless, the ZB kit may require further optimization since the A260/A230 ratios reported in other studies comparing the performances of several commercial kits for bacterial DNA extraction from different sample types were all within the expected range [22, 54, 55]. On the other hand, the extraction process with the BT kit tended to acquire higher levels of contaminating bacteria resulting in 16S RNA gene copy cut-off values that were 2 to 3 orders of magnitude higher compared to the ZB kit. We speculate that the minimal steps and low bioburden reagents in the ZB kit contributed to lower 16S copies found in the negative extraction controls.

Our protocol for 16S rRNA sequencing on the Illumina platform calls for sample normalization to 5 × 10^5^ 16S rRNA copies/ 3μL before PCR amplification and library preparation to have a 10X target sequencing coverage. This normalization appeared to eliminate between-kit differences in the number of 16S rRNA gene sequences (Fig 3B). Studies evaluating kit effects on bacterial diversity typically focus on the differences observed in the concentration of 16S rRNA gene copies detected via quantitative PCR without paying much attention to the actual number of 16S rRNA gene sequences recovered [54, 56]. The contribution of unnormalized DNA concentrations to the observed difference in microbiota profile between kits needs to be clarified. Furthermore, the number of non-16S rRNA gene sequences, most of which were derived from the host (Fig 4A), accounted for 18% and 24% of total sequences obtained through BT and ZB kit extraction processes, respectively, confirming that the 16S rRNA gene primer set used in this study was highly specific and PCR thermocycling conditions were optimal.

In addition to pre-sequencing data, bacterial compositions and diversity metrics are commonly used as proxies for performance in extraction kit-comparison studies [22, 55, 57, 58]. Here, all the predominant ASVs detected in samples extracted with one kit were present in replicate samples extracted with the other kit and did not show significant differences at the genus level (Tables 3 and 4 and Fig 5). Probing further into shared taxa at higher levels of taxonomic resolution, a total of 11 genus level and 2 family level taxa were differentially abundant in one or more sample types (Table 4). Although majority of these differentially abundant taxa were low in relative abundance, *Vibrio* was more prevalent in samples extracted with the BT kit, which is intriguing since gram-negative bacteria are easy to lyse [59], and should therefore be represented at similar levels in samples extracted with either kit. However, between-kits differences in alpha and beta diversity metrics were statistically indistinguishable (Figs 6 and 7). The high variability in the weighted UniFrac distances reflects differential abundances of several taxa between the kits (Table 4, S1 File).

Other studies have typically compared multiple extraction kits using a single sample type [22, 55, 57, 58]. In this study, although post-sequencing analysis revealed comparable performances between the BT and ZB kits across multiple types of respiratory samples, the impact of sample type on other DNA extraction methods requires further investigation. For example, between-kit differences appear to be magnified in gut samples [54, 60] and reduced in respiratory samples [56, 57], possibly due to body site differences in microbial densities and other factors such as PCR inhibitors, complexity of matrix, high bacteria density and inhibitors present in the sample. Nevertheless, both BT and ZB kits performed similarly in terms of extracting specific bacteria in the mock community and showed differences in bacterial compositions and diversity metrics between different respiratory sites (Tables 2, 3), and have therefore validated our previous reports [2, 15–17]. While high numbers of unique ASVs were present in samples extracted with either kit, their total relative abundances were very low. The presence of unique ASVs in low biomass samples, such as respiratory samples, is not unexpected [23, 24]. However, since there is no standard reference for avian respiratory microbiome, we cannot determine whether the unique ASVs are contaminants despite multiple steps of filtering, or rare taxa inhabiting the avian respiratory tract [61–63]. We suggest that these low abundance taxa be investigated further using a combination of culture dependent and independent approaches. With that said, the ZB kit is less likely to contain contaminant bacterial DNA, thereby reducing the likelihood of excluding samples with low DNA concentrations from analysis. Note that while both kits performed similarly in the detection of bacterial taxa from different sample types, the number of ASVs from live choanal swab were exaggerated with the BT kit (Fig 6A), suggesting that this kit is not optimal for this sample type.

The design of this study resulted in uneven distribution of sample replicates between sample types. For each kit, there were 8 replicates/sample type for lavage and nasal wash samples and 4 replicates/sample type for tracheal wash and swab samples. Therefore, reasonable comparisons between sample types within each kit could not be achieved in this study.

In conclusion, both the ZB and BT methods of DNA extraction produced similar chicken respiratory microbiome data in terms of bacterial diversity and taxonomic profiles.

## Supporting information

S1 File Data workbook. DESeq2 analysis for shared taxa within a sample type between kits.
(XLSX)

S2 File Data workbook. Average relative abundance of shared and unshared taxa within a sample type between kits.
(XLSX)

## References

[1] Simon K, Verwoolde MB, Zhang J, Smidt H, de Vries Reilingh G, Kemp B, et al Long-term effects of early life microbiota disturbance on adaptive immunity in laying hens. Poult Sci. 2016;95: 1543–1554. 10.3382/ps/pew088 26976906

[2] Johnson TJ, Youmans BP, Noll S, Cardona C, Evans NP, Karnezos TP, et al A Consistent and Predictable Commercial Broiler Chicken Bacterial Microbiota in Antibiotic-Free Production Displays Strong Correlations with Performance. Appl Environ Microbiol. 2018;84 10.1128/AEM.00362-18 29625981 PMC5981067

[3] Torok VA, Hughes RJ, Mikkelsen LL, Perez-Maldonado R, Balding K, MacAlpine R, et al Identification and Characterization of Potential Performance-Related Gut Microbiotas in Broiler Chickens across Various Feeding Trials. Appl Environ Microbiol. 2011;77: 5868–5878. 10.1128/AEM.00165-11 21742925 PMC3165380

[4] Nava GM, Bielke LR, Callaway TR, Castañeda MP. Probiotic alternatives to reduce gastrointestinal infections: the poultry experience. Anim Health Res Rev. 2005;6: 105–118. 10.1079/ahr2005103 16164012

[5] Stanley D, Denman SE, Hughes RJ, Geier MS, Crowley TM, Chen H, et al Intestinal microbiota associated with differential feed conversion efficiency in chickens. Appl Microbiol Biotechnol. 2012;96: 1361–1369. 10.1007/s00253-011-3847-5 22249719

[6] Costea PI, Zeller G, Sunagawa S, Pelletier E, Alberti A, Levenez F, et al Towards standards for human fecal sample processing in metagenomic studies. Nat Biotechnol. 2017;35: 1069–1076. 10.1038/nbt.3960 28967887

[7] Sinha R, Abu-Ali G, Vogtmann E, Fodor AA, Ren B, Amir A, et al Assessment of variation in microbial community amplicon sequencing by the Microbiome Quality Control (MBQC) project consortium. Nature Biotechnology. 2017;35: 1077–1086. 10.1038/nbt.3981 28967885 PMC5839636

[8] Koren O, Knights D, Gonzalez A, Waldron L, Segata N, Knight R, et al A Guide to Enterotypes across the Human Body: Meta-Analysis of Microbial Community Structures in Human Microbiome Datasets. PLOS Computational Biology. 2013;9: e1002863 10.1371/journal.pcbi.1002863 23326225 PMC3542080

[9] Brooks JP, Edwards DJ, Harwich MD, Rivera MC, Fettweis JM, Serrano MG, et al The truth about metagenomics: quantifying and counteracting bias in 16S rRNA studies. BMC Microbiol. 2015;15: 66 10.1186/s12866-015-0351-6 25880246 PMC4433096

[10] Schirmer M, Ijaz UZ, D’Amore R, Hall N, Sloan WT, Quince C. Insight into biases and sequencing errors for amplicon sequencing with the Illumina MiSeq platform. Nucleic Acids Res. 2015;43: e37 10.1093/nar/gku1341 25586220 PMC4381044

[11] Bag S, Saha B, Mehta O, Anbumani D, Kumar N, Dayal M, et al An Improved Method for High Quality Metagenomics DNA Extraction from Human and Environmental Samples. Sci Rep. 2016;6: 26775 10.1038/srep26775 27240745 PMC4886217

[12] Baker GC, Smith JJ, Cowan DA. Review and re-analysis of domain-specific 16S primers. J Microbiol Methods. 2003;55: 541–555. 10.1016/j.mimet.2003.08.009 14607398

[13] Cruaud P, Vigneron A, Lucchetti-Miganeh C, Ciron PE, Godfroy A, Cambon-Bonavita M-A. Influence of DNA Extraction Method, 16S rRNA Targeted Hypervariable Regions, and Sample Origin on Microbial Diversity Detected by 454 Pyrosequencing in Marine Chemosynthetic Ecosystems. Appl Environ Microbiol. 2014;80: 4626–4639. 10.1128/AEM.00592-14 24837380 PMC4148798

[14] Tremblay J, Singh K, Fern A, Kirton ES, He S, Woyke T, et al Primer and platform effects on 16S rRNA tag sequencing. Front Microbiol. 2015;6 10.3389/fmicb.2015.00771 26300854 PMC4523815

[15] Ngunjiri JM, Taylor KJM, Abundo MC, Jang H, Elaish M, Kc M, et al Farm Stage, Bird Age, and Body Site Dominantly Affect the Quantity, Taxonomic Composition, and Dynamics of Respiratory and Gut Microbiota of Commercial Layer Chickens. Appl Environ Microbiol. 2019;85 10.1128/AEM.03137-18 30824436 PMC6495750

[16] Taylor KJM, Ngunjiri JM, Abundo MC, Jang H, Elaish M, Ghorbani A, et al Respiratory and Gut Microbiota in Commercial Turkey Flocks with Disparate Weight Gain Trajectories Display Differential Compositional Dynamics. Appl Environ Microbiol. 2020;86 10.1128/AEM.00431-20 32276973 PMC7267191

[17] Abundo MC, Ngunjiri JM, Taylor KJM, Ji H, Ghorbani A, K C M, et al Evaluation of Sampling Methods for the Study of Avian Respiratory Microbiota. Avian Diseases. 2020;In Press. 10.1637/aviandiseases-D-19-00200 33205170

[18] Vermassen A, Leroy S, Talon R, Provot C, Popowska M, Desvaux M. Cell Wall Hydrolases in Bacteria: Insight on the Diversity of Cell Wall Amidases, Glycosidases and Peptidases Toward Peptidoglycan. Front Microbiol. 2019;10 10.3389/fmicb.2019.00331 30873139 PMC6403190

[19] Shehadul Islam M, Aryasomayajula A, Selvaganapathy PR. A Review on Macroscale and Microscale Cell Lysis Methods. Micromachines (Basel). 2017;8 10.3390/mi8030083

[20] Yu Z, Morrison M. Improved extraction of PCR-quality community DNA from digesta and fecal samples. BioTechniques. 2004;36: 808–812. 10.2144/04365ST04 15152600

[21] Liu Z, Lozupone C, Hamady M, Bushman FD, Knight R. Short pyrosequencing reads suffice for accurate microbial community analysis. Nucleic Acids Res. 2007;35: e120 10.1093/nar/gkm541 17881377 PMC2094085

[22] Sohrabi M, Nair RG, Samaranayake LP, Zhang L, Zulfiker AHM, Ahmetagic A, et al The yield and quality of cellular and bacterial DNA extracts from human oral rinse samples are variably affected by the cell lysis methodology. J Microbiol Methods. 2016;122: 64–72. 10.1016/j.mimet.2016.01.013 26812577

[23] Salter SJ, Cox MJ, Turek EM, Calus ST, Cookson WO, Moffatt MF, et al Reagent and laboratory contamination can critically impact sequence-based microbiome analyses. BMC Biology. 2014;12: 87 10.1186/s12915-014-0087-z 25387460 PMC4228153

[24] Karstens L, Asquith M, Davin S, Fair D, Gregory WT, Wolfe AJ, et al Controlling for Contaminants in Low-Biomass 16S rRNA Gene Sequencing Experiments. mSystems. 2019;4 10.1128/mSystems.00290-19 31164452 PMC6550369

[25] Shabbir MZ, Malys T, Ivanov YV, Park J, Shabbir MAB, Rabbani M, et al Microbial communities present in the lower respiratory tract of clinically healthy birds in Pakistan. Poult Sci. 2015;94: 612–620. 10.3382/ps/pev010 25667427 PMC6281309

[26] Glendinning L, McLachlan G, Vervelde L. Age-related differences in the respiratory microbiota of chickens. PLoS One. 2017;12 10.1371/journal.pone.0188455 29166670 PMC5699826

[27] Sohail MU, Hume ME, Byrd JA, Nisbet DJ, Shabbir MZ, Ijaz A, et al Molecular analysis of the caecal and tracheal microbiome of heat-stressed broilers supplemented with prebiotic and probiotic. Avian Pathol. 2015;44: 67–74. 10.1080/03079457.2015.1004622 25564364

[28] Kursa O, Tomczyk G, Sawicka-Durkalec A, Giza A, Słomiany-Szwarc M. Characterization of the upper respiratory tract microbiome of turkeys. In Review; 2020 Jun. 10.21203/rs.3.rs-33858/v1 PMC7843632 33510238

[29] DNeasy® Blood & Tissue Handbook July 2020. https://www.qiagen.com/us/resources/download.aspx?id=68f29296-5a9f-40fa-8b3d-1c148d0b3030&lang=en2020 [cited 27 Aug 2020]. Available: https://www.qiagen.com/us/resources/download.aspx?id=68f29296-5a9f-40fa-8b3d-1c148d0b3030&lang=en

[30] ZymoBIOMICS^TM^ DNA Miniprep Kit Instruction Manual ver 1.4.1. 27 Aug 2020 [cited 27 Aug 2020]. Available: https://files.zymoresearch.com/protocols/_d4300t_d4300_d4304_zymobiomics_dna_miniprep_kit.pdf

[31] Caporaso JG, Lauber CL, Walters WA, Berg-Lyons D, Huntley J, Fierer N, et al Ultra-high-throughput microbial community analysis on the Illumina HiSeq and MiSeq platforms. The ISME Journal. 2012;6: 1621–1624. 10.1038/ismej.2012.8 22402401 PMC3400413

[32] Gilbert Jack A., Meyer Folker. Modeling the Earth microbiome. Microbe. 2012;7: 64–69.

[33] Gohl DM, Vangay P, Garbe J, MacLean A, Hauge A, Becker A, et al Systematic improvement of amplicon marker gene methods for increased accuracy in microbiome studies. Nature Biotechnology. 2016;34: 942–949. 10.1038/nbt.3601 27454739

[34] Bushnell B, Rood J, Singer E. BBMerge–Accurate paired shotgun read merging via overlap. PLOS ONE. 2017;12: e0185056 10.1371/journal.pone.0185056 29073143 PMC5657622

[35] Martin M. Cutadapt removes adapter sequences from high-throughput sequencing reads. EMBnet.journal. 2011;17: 10–12. 10.14806/ej.17.1.200

[36] Bolyen E, Rideout JR, Dillon MR, Bokulich NA, Abnet CC, Al-Ghalith GA, et al Reproducible, interactive, scalable and extensible microbiome data science using QIIME 2. Nature Biotechnology. 2019;37: 852–857. 10.1038/s41587-019-0209-9 31341288 PMC7015180

[37] Callahan BJ, McMurdie PJ, Rosen MJ, Han AW, Johnson AJA, Holmes SP. DADA2: High resolution sample inference from Illumina amplicon data. Nature methods. 2016;13: 581 10.1038/nmeth.3869 27214047 PMC4927377

[38] Rognes T, Flouri T, Nichols B, Quince C, Mahé F. VSEARCH: a versatile open source tool for metagenomics. PeerJ. 2016;4 10.7717/peerj.2584 27781170 PMC5075697

[39] Quast C, Pruesse E, Yilmaz P, Gerken J, Schweer T, Yarza P, et al The SILVA ribosomal RNA gene database project: improved data processing and web-based tools. Nucleic Acids Research. 2013;41: D590 10.1093/nar/gks1219 23193283 PMC3531112

[40] Bokulich NA, Kaehler BD, Rideout JR, Dillon M, Bolyen E, Knight R, et al Optimizing taxonomic classification of marker-gene amplicon sequences with QIIME 2’s q2-feature-classifier plugin. Microbiome. 2018;6: 90 10.1186/s40168-018-0470-z 29773078 PMC5956843

[41] Lozupone CA, Hamady M, Kelley ST, Knight R. Quantitative and Qualitative β Diversity Measures Lead to Different Insights into Factors That Structure Microbial Communities. Applied and Environmental Microbiology. 2007;73: 1576 10.1128/AEM.01996-06 17220268 PMC1828774

[42] Anderson MJ. Permutational Multivariate Analysis of Variance (PERMANOVA). Wiley StatsRef: Statistics Reference Online. American Cancer Society; 2017 pp. 1–15. 10.1002/9781118445112.stat07841

[43] DeSantis TZ, Hugenholtz P, Larsen N, Rojas M, Brodie EL, Keller K, et al Greengenes, a Chimera-Checked 16S rRNA Gene Database and Workbench Compatible with ARB. Appl Environ Microbiol. 2006;72: 5069–5072. 10.1128/AEM.03006-05 16820507 PMC1489311

[44] Pielou EC. The measurement of diversity in different types of biological collections. Journal of Theoretical Biology. 1966;13: 131–144. 10.1016/0022-5193(66)90013-0

[45] Nahm FS. Nonparametric statistical tests for the continuous data: the basic concept and the practical use. Korean J Anesthesiol. 2016;69: 8–14. 10.4097/kjae.2016.69.1.8 26885295 PMC4754273

[46] R Core Team. R: A Language and Environment for Statistical Computing. R Foundation for Statistical Computing; 2018 Available: https://www.r-project.org/

[47] Love MI, Huber W, Anders S. Moderated estimation of fold change and dispersion for RNA-seq data with DESeq2. Genome Biology. 2014;15: 550 10.1186/s13059-014-0550-8 25516281 PMC4302049

[48] Davis NM, Proctor DM, Holmes SP, Relman DA, Callahan BJ. Simple statistical identification and removal of contaminant sequences in marker-gene and metagenomics data. Microbiome. 2018;6: 226 10.1186/s40168-018-0605-2 30558668 PMC6298009

[49] Zymo Research. Certificate of Analysis. 21 Aug 2020 [cited 21 Aug 2020]. Available: https://s3.amazonaws.com/cofaman/cofaman/D6300_ZRC190633CofA.pdf

[50] ZymoBIOMICS^TM^ Microbial Community Standard Catalog No. D6300. 27 Aug 2020 [cited 27 Aug 2020]. Available: https://files.zymoresearch.com/protocols/_d6300_zymobiomics_microbial_community_standard.pdf

[51] Koetsier G, Cantor E. A Practical Guide to Analyzing Nucleic Acid Concentration and Purity with Microvolume Spectrophotometers. New England BioLabs: Technical note. 2019; 8.

[52] Glasel J. Validity of Nucleic Acid Purities Monitored by A260/A280 Absorbance Ratios. BioTechniques. 1995; 62–63. 7702855

[53] Laws GM, Adams SP. Measurement of 8-OHdG in DNA by HPLC/ECD: The Importance of DNA Purity. BioTechniques. 1996;20: 36–38. 10.2144/96201bm06 8770401

[54] Pankoke H, Maus I, Loh G, Hüser A, Seifert J, Tilker A, et al F5Evaluation of commercially available DNA extraction kits for the analysis of the broiler chicken cecal microbiota. FEMS Microbiology Letters. 2019 [cited 28 Aug 2020]. 10.1093/femsle/fnz033 30915459 PMC8112482

[55] Teng F, Darveekaran Nair SS, Zhu P, Li S, Huang S, Li X, et al Impact of DNA extraction method and targeted 16S-rRNA hypervariable region on oral microbiota profiling. Scientific Reports. 2018;8: 16321 10.1038/s41598-018-34294-x 30397210 PMC6218491

[56] Pérez-Losada M, Crandall KA, Freishtat RJ. Comparison of two commercial DNA extraction kits for the analysis of nasopharyngeal bacterial communities. microbiology 2016, Vol 2, Pages 108–119. 2016 [cited 29 Jun 2020]. 10.3934/microbiol.2016.2.108

[57] Terranova L, Oriano M, Teri A, Ruggiero L, Tafuro C, Marchisio P, et al How to Process Sputum Samples and Extract Bacterial DNA for Microbiota Analysis. Int J Mol Sci. 2018;19 10.3390/ijms19103256 30347804 PMC6214103

[58] Lazarevic V, Gaïa N, Girard M, François P, Schrenzel J. Comparison of DNA Extraction Methods in Analysis of Salivary Bacterial Communities. PLOS ONE. 2013;8: e67699 10.1371/journal.pone.0067699 23844068 PMC3701005

[59] Silhavy TJ, Kahne D, Walker S. The Bacterial Cell Envelope. Cold Spring Harbor Perspectives in Biology. 2010;2 10.1101/cshperspect.a000414 20452953 PMC2857177

[60] Lim MY, Song E-J, Kim SH, Lee J, Nam Y-D. Comparison of DNA extraction methods for human gut microbial community profiling. Systematic and Applied Microbiology. 2018;41: 151–157. 10.1016/j.syapm.2017.11.008 29305057

[61] Kaminsky R, Morales SE. Conditionally Rare Taxa Contribute but Do Not Account for Changes in Soil Prokaryotic Community Structure. Front Microbiol. 2018;9 10.3389/fmicb.2018.00809 29755437 PMC5934496

[62] Pedrós-Alió C. Dipping into the Rare Biosphere. Science. 2007;315: 192–193. 10.1126/science.1135933 17218512

[63] Lynch MDJ, Neufeld JD. Ecology and exploration of the rare biosphere. Nature Reviews Microbiology. 2015;13: 217–229. 10.1038/nrmicro3400 25730701

